# Member Perceptions of the One Health Initiative at a Zoological Institution

**DOI:** 10.3389/fvets.2018.00022

**Published:** 2018-02-26

**Authors:** Hannah Padda, Amy Niedbalski, Erin Tate, Sharon L. Deem

**Affiliations:** ^1^Brown School, Washington University in St. Louis, St. Louis, MO, United States; ^2^Institute for Conservation Medicine, Saint Louis Zoo, St. Louis, MO, United States; ^3^Department of Audience Research, Saint Louis Zoo, St. Louis, MO, United States

**Keywords:** conservation medicine, emerging diseases, human health, Saint Louis Zoo, public health, transdisciplinary

## Abstract

Zoological institutions play an important role in promoting the goals of the One Health movement. We launched the Institute for Conservation Medicine (ICM) at the Saint Louis Zoo in 2011 to advance the goals of One Health. In 2016, we distributed a survey to Zoo members to evaluate member awareness and understanding of One Health and to provide direction for future communication and actions from the ICM. We hypothesized that Zoo members would be aware of One Health and care about infectious disease issues. Survey results showed Zoo members primarily cared about chronic, non-infectious diseases and their associated economic costs, with participants ranking their top three health issues of concern for humans as nutrition/obesity/diet (49%), costs of health care (48%), and cancer (37%). Zoo members were interested in the roles of zoos in One Health and found them important, but were less aware of the Saint Louis Zoo’s actions that did not directly relate to animal welfare. Only 6% of members had awareness of the term “One Health” and 16% were aware of the term “Conservation Medicine.” These results suggest that zoos may do better to tailor One Health messaging to align with member interests. Messaging and programming from the Saint Louis Zoo will now include the direct benefits to human health that zoos offer, in addition to the ICM’s more ecologically focused activities. This study offered valuable insight into how Zoo members view One Health and may serve as a template to help zoological institutions develop and promote One Health.

## Introduction

Worldwide, there has been a shift in disease research and interests from infectious, communicable diseases to non-communicable, chronic, human-induced diseases (e.g., obesity, diabetes, heart disease), as these non-infectious diseases increasingly threaten human health and overall public health (1). This shift, termed the “epidemiologic transition,” describes the changes in patterns of population age distributions, mortality, fertility, life expectancy, and causes of death (1, 2). The epidemiologic transition is associated with industrialization and urbanization, with impacts on the environment and all living things within it. As such, chronic, non-communicable diseases are at the forefront of health care for humans, and these diseases result in increasing health-care costs and are strongly associated with obesity. Depression, for example, is the second leading cause of disability adjusted life years worldwide (3).

During this time of shifting human health challenges, conservation challenges have also increased. Since the 1970s, average global population sizes of wildlife have decreased by more than 50%, leading to the development of the field of conservation medicine in the 1990s (4–7). This is a holistic approach that evolved at a time when infectious and non-infectious diseases increasingly threatened the conservation of wildlife, wild lands, and the humans dependent on both (4). While conservation medicine provides a broad approach to the ecological context of health, a newer movement has emerged in response to the increase in emerging infectious diseases and their often zoonotic origins (8, 9). This initiative, most recently named One Health, has been defined as “a transdisciplinary approach to sustainable management of complex health problems arising from the interaction of animals, humans, and their environment” (10). The One Health initiative may best be considered from its two core components: translational, focusing on comparative medicine, and ecological.

One Health initiatives from zoos have primarily focused on research and messaging about zoonotic diseases and the human–wildlife interface (8, 11–13). Communicating to people about these threats to both human and wildlife health is an important part of One Health (11). One goal of these communications has been to generate public support for healthy wildlife populations with the assumption that if disease risks are well communicated, then public attitudes toward zoonotic disease management should be impacted (11, 14). As the field of One Health grows and public awareness increases, broader communications about the role of One Health are necessary, and zoological institutions should be at the forefront of the One Health movement, because of the transdisciplinary nature of their work and their strong focus on recreation, education, research, and conservation (8, 12, 15). Recreation is of increasing importance in the context of One Health because of the health benefits for zoo visitors in a largely urbanized and sedentary world. On average, Americans spend 87% of their time indoors, and 6% of their time in enclosed vehicles, which has drastic impacts on human health (16). Zoos connect people with nature and encourage healthy human habits through physical movement across zoo campuses and interactions with exhibits and animals (17, 18).

Zoos have a unique position within One Health, for which we have identified seven important tenets. These include (1) providing health care for zoo wildlife to help sustain biodiversity; (2) studies on diseases of conservation concern; (3) understanding diseases in zoo wildlife as sentinels for emerging diseases of humans and other animals; (4) surveillance of disease in wild animals at the interface of wildlife, domestic animals, and humans; (5) contributing to the field of comparative medicine and the discovery of all life forms; (6) exploration of the diversity of life at both micro and macro levels; and (7) human health benefits from nature (5, 8, 18). These roles inform the actions that zoos take in regards to One Health messaging and encourage zoos to invest resources into promoting One Health to visitors, members, and communities.

In linking the roles of zoos in One Health and current public health issues, it has been demonstrated that exposure to nature for at least 5 h a month has lasting restorative effects, such as elevating mood and decreasing risk of depression (10). Studies have indicated that public health strategies should address these concerns *via* an upstream approach that provides opportunities for people to connect with nature, such as spending time in greenspaces, including zoos (19–21). Nature can promote health through many different pathways, but most broadly does so through environmental conditions, physiological and psychological states, and behaviors (17). We also know that children with attention-deficit disorders exhibit diminished symptoms following activities in green settings, such as zoos (22). Finally, it has been shown that zoos may improve both psychological and physiological well-being through decreasing stress and increasing physical activity (18, 23).

In response to the growing conservation and public health challenges, the Saint Louis Zoo launched the Institute for Conservation Medicine (ICM) in 2011 to advance the goals of One Health. The ICM’s mission statement reads as follows, “The Saint Louis Zoo Institute for Conservation Medicine takes a holistic approach to research on wildlife, public health and sustainable ecosystems to ensure healthy animals and healthy people.” The 50,000 ± Saint Louis Zoo member households, which equates to more than one-third of total annual Zoo visitation, often receive One Health messaging. This includes a quarterly magazine, often with a feature on the ICM’s activities, and weeks prior to the Zoo’s annual One Health Fair a promotional email is distributed to all Zoo members (24). Zoo members also consistently report being significantly more aware of the Zoo’s conservation work in Missouri and around the world than those Zoo visitors who are not members [Niedbalski, unpublished data]. Additionally, the Zoo’s social media accounts, which have nearly half a million followers, provide occasional updates on One Health activities and the ICM.

While the ICM was founded over 5 years ago, it had yet to be evaluated whether the presence of ICM in our newsletters, social media and other layperson friendly publications, and zoo-sponsored activities was having an impact on Zoo goers’ understanding of One Health. The primary objective of this study was to evaluate Zoo member understanding of the concept of One Health 5 years after the establishment of the ICM to provide department evaluation and direction for the coming 5 years. Additionally, we wished to determine what health concerns/issues were at the forefront of Zoo members’ thinking.

Our hypotheses were that Zoo members would have a high level of awareness regarding One Health and that they would be aware of and care about infectious disease issues, since the survey was administered during significant media coverage of the spread of the Zika virus in North America. Therefore, we asked respondents’ awareness of types of One Health projects in which the Zoo is involved, rating of concern of zoonotic disease transmission (with an open-ended follow-up response), and awareness and explanation of the terms One Health and Conservation Medicine. Finally, we asked ratings of awareness, interest, and importance of five of the various roles of the Zoo’s involvement in One Health areas.

## Materials and Methods

We distributed an online survey *via* email to 2,983 Zoo members selected from the member database in June 2016 (Table 1). Zoo members were defined as individuals or households who purchased an annual membership through the Saint Louis Zoo. The Saint Louis Zoo maintains a database of approximately 50,000 member households, with just over 30,000 providing permission to utilize their email addresses for receiving communication from the Zoo. The Audience Research department of the Saint Louis Zoo provides data-driven evidence for informed decision-making that contributes to the achievement of the Saint Louis Zoo’s mission-oriented goals. Audience Research staff at the Zoo sends approximately 10 member surveys per year, with no member household receiving more than one survey in a 365-day period. Therefore, email addresses are subset into 10 smaller samples (of approximately 3,000 email addresses), and each subset is representative of the total member population based on membership level. This study involved an online survey of members and was exempt from Institutional Review Board approval. Completion of the survey indicated consent.

**Table 1:** Items on the One Health questionnaire answered by survey participants in the 2016 One Health membership survey at the Saint Louis Zoo.

Survey questions					
What do you think are currently the most important health issues facing humans? Please select your top THREE choices. 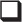 Nutrition/obesity/diet 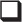 Substance abuse (tobacco, opioids, alcohol, etc.) 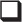 Access to health care, environmental quality (water, soil, etc.) 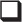 Mental health 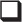 Wildlife-related diseases (Zika, Rabies, etc.) 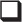 HIV/AIDS 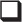 Cancer 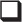 Heart disease 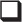 Diabetes 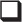 Costs of health care/insurance 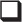 Climate change (extreme weather, etc.) 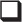 Population growth 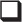 None of these 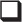 Other (please specify)					

2. Which ONE of these issues are you most concerned about in the future? 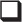 Nutrition/obesity/diet 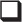 Substance abuse (tobacco, opioids, alcohol, etc.) 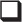 Access to health care, environmental quality (water, soil, etc.) 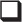 Mental health 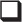 Wildlife-related diseases (Zika, Rabies, etc.) 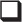 HIV/AIDS 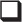 Cancer 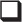 Heart disease 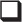 Diabetes 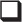 Costs of health care/insurance 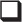 Climate change (extreme weather, etc.) 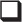 Population growth 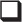 None of these 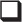 (Insert text from other)					

3. Why? (Referencing question 2)					

4. Are you aware that the Saint Louis Zoo conducts projects (science, outreach, etc.) involving the following areas?					
		Yes		No	
			
Animal health		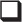		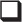	
Human health		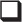		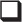	
Environmental health		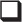		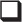	

5. Please describe any of the projects with which you may be familiar.					

6. How concerned are you about the following groups contracting diseases from wildlife?					
	Not at all concerned	Not too concerned	Neutral	Somewhat concerned	Very concerned
	
Humans	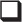	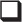	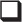	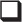	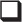
Domestic animals	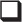	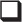	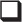	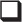	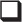

7. Why or why not?					

8. Have you ever heard either of the following terms?					
		Yes		No	
			
One Health		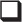		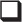	
Conservation medicine		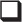		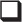	

9. What do/does the term(s) mean to you?					

10. Please review the following five roles that the Saint Louis Zoo has in this area. Please rate your AWARENESS that the Zoo is involved in each role, your INTEREST that the Zoo is involved in each role, and the IMPORTANCE you place on the Zoo’s involvement in each role.					
1 = not at all, 5 = completely					
Providing health care to zoo animals					
Awareness	1	2	3	4	5
Interest	1	2	3	4	5
Importance	1	2	3	4	5
Research on how disease can threaten species with extinction					
Awareness	1	2	3	4	5
Interest	1	2	3	4	5
Importance	1	2	3	4	5
Studying disease in animals in zoo care					
Awareness	1	2	3	4	5
Interest	1	2	3	4	5
Importance	1	2	3	4	5
Studying how diseases in wild animals have an impact on domestic animals and humans					
Awareness	1	2	3	4	5
Interest	1	2	3	4	5
Importance	1	2	3	4	5
Research comparing diseases between different species, including humans					
Awareness	1	2	3	4	5
Interest	1	2	3	4	5
Importance	1	2	3	4	5

11. With which gender do you most closely identify? Male 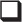 Female 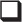					

12. Which of the following categories includes your age?					
18–24 years 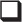 25–34 years 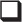 35–44 years 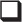 45–54 years 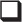 55–64 years 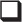 65+ years 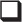					

13. What is your five digit ZIP code?					

14. Do you have any additional comments for the Zoo?					

We used IBM SPSS Version 24 to analyze the data and run descriptive statistics. We obtained data on median household income from https://www.incomebyzipcode.com and matched it to each respondent’s zip code. These data were dichotomized into higher or lower than the median household income in the United States, which the census reported as $56,516 (25). For the open-ended questions, two individuals (HP and ET) sorted and independently coded the qualitative data into different categories, and then a third individual (AN) assessed and made the final decision on the coding when there were discrepancies between the original two coders. Survey answers were stratified by age, gender, and income category and then analyzed in a crosstab analysis. We collapsed age categories into three categories for the crosstab analysis: 18–34, 35–54, and 55+.

## Results

### Sample

There were 439 survey respondents. We excluded 154 responses, as they were incomplete, leaving 285 completed surveys, for a response rate of 9.6%. Eighty percent (228) of respondents identified as females and 20% (57) identified as males. Sixty-two percent of those surveyed live in zip codes above the United States median household income. The majority of respondents were over the age of 35, with the largest age groups representing those between the ages of 35 and 44 (28%) and over the age of 65 (22%) (Table 2).

**Table 2:** Demographic characteristics of the four populations of Saint Louis Zoo patrons: those surveyed during the 2016 One Health Study at the Saint Louis Zoo, Saint Louis Zoo members, Saint Louis Zoo visitors, and people who live in the Zoo Museum District^a^.

**Demographics**				

	Study group (%)	Zoo members (%)	Zoo visitors (%)	Zoo-museum district^a^ (%)
**Gender**				
Male	20	30	30	52
Female	80	70	70	48

**Age**				
18–24	1	0	19	12
25–34	14	14	33	18
35–44	28	29	22	15
45–54	15	13	14	17
55–64	20	20	9	18
65+	22	24	3	20

**Income**				
Less than $25,000	1	1	12	24
$25,000–49,999	18	9	27	23
$50,000–$74,999	54	19	20	17
$75,000–$149,999	25	49	33	25
$150,000 or more	2	22	8	11

*^a^Zoo Museum District is the tax district from which the Saint Louis Zoo receives taxpayer support*.

### Survey Results

We asked participants to select and rank the top three health issues facing humans from an extensive predefined list of choices, which included an “other” option, allowing respondents to provide an answer that was not already in the list. Participants identified the top three important health issues facing humans as (1) nutrition/obesity/diet (49%), (2) costs of health care (48%), and (3) cancer (37%). These responses held true for future concerns, in which participants identified the one issue about which they were most concerned for the future, with costs of health care, nutrition/obesity/diet, and cancer in the top three, at 2, 16, and 11%, respectively. Only 1% of respondents were very concerned about wildlife-related diseases being a future health threat. These responses only differed significantly for the answer choice “mental health,” with 24 females identifying mental health as an important health issue compared to 0 males. Results did not vary significantly by age category or income level. When respondents were asked why they selected the one issue they identified as being the most important for the future, most responses referenced the cost of health care, Earth’s limited resources, and the broad scope of the one issue they chose (Table 3).

**Table 3:** A sample of representative quotes from responses to a One Health membership survey distributed in 2016 at the Saint Louis Zoo.

Quotes
3. What do you think are currently the most important health issues facing humans? Which one of these issues are you most concerned about in the future? Why?
“If we cannot sustain a livable environment, none of this matters.”
“Environmental factors contribute to many of the other issues we face, from cancer to health risks, we don’t even know about.”
“With the changes in our health-care system recently, I am finding that even as a middle class American, health care is expensive. […] Cost should not be the driving factor in our health care.”
7. How concerned are you about humans contracting diseases from wildlife? How concerned are you about domestic animals contracting diseases from wildlife? Why?
“Although humans do contract diseases from animals (ebola, strains of influenza, and even originally HIV), there are many other health concerns that lead to more human diseases and deaths that are purely human caused. Plus, our human population is still increasing dramatically.”
“In most cases, humans don’t have much actual contact with wildlife except for insects—the zeka (sp) virus is still an unknown. In spite of certain special interest groups, I do not see a threat to domestic animals (except for the occasional dog vs skunk—or, more seriously, snake)”
“Not in the news so assuming it’s not a huge issue”
9. What do the term(s) One Health and/or Conservation medicine mean to you?
“Working with the environment. The health of everything on earth is interconnected”
“Simultaneously addressing the combined health concerns of humans and wildlife.”
“One Health means nothing to me. Conservation medicine means (to me) ways to keep animals healthy so they can survive and reproduce, particularly for endangered species.”

*In this table, all the questions refer to survey questions from Table 1*.

Eighty-six percent of respondents were aware that the Saint Louis Zoo conducts projects involving animal health, but only 22% were aware of the Zoo’s involvement with projects for human health, and 61% were aware of projects regarding environmental health. However, the majority of those who claimed to be aware were not able to name a specific project or program when asked to describe them. Fifty percent of participants were concerned about humans contracting diseases from wildlife, 34% were not concerned, and the remaining 16% were neutral about the issue. We asked participants to provide why they were, or were not, concerned about humans contracting diseases from wildlife. Among those who were not concerned, 40% “just don’t think about it” and 21% have no or minimal interaction with wildlife (Table 3). Participants also answered questions about their concern for domestic animals contracting diseases from wildlife. Forty-eight percent were concerned about domestic animals contracting diseases from wildlife, 33% were not concerned, and 19% were neutral about animals contracting disease from wildlife. Of those who were not concerned, 36% “just don’t think about it,” while 23% indicated minimal interactions with wildlife (Table 3). Only 6% of survey participants had heard of the term “One Health,” whereas 16% had heard the term “conservation medicine.” Participants also provided their interpretation of the term(s) “One Health” and “conservation medicine,” and these responses focused on how “the health of everything on earth is interconnected” (Table 3).

**Figure 1:**
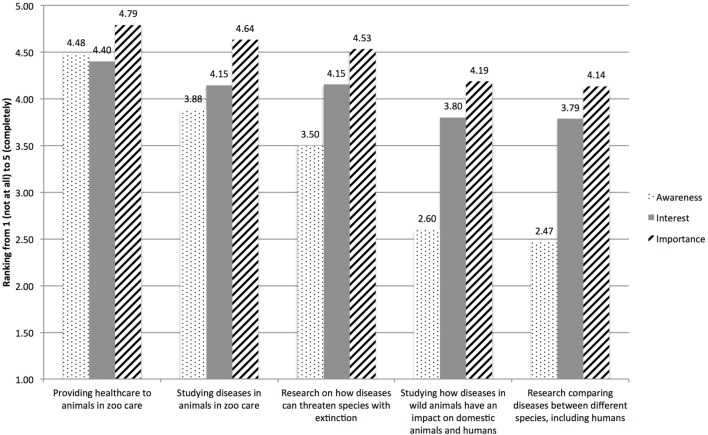
Saint Louis Zoo member responses from the One Health membership survey on the five key tenets that zoological institutions have in regards to One Health. Awareness, interest, and importance of these actions at the Saint Louis Zoo were measured for each tenet.

We asked participants to rank their awareness of the Saint Louis Zoo’s role in five different areas of One Health, their interest that the Zoo participates in these areas, and the importance of the Zoo’s participation (Figure 1). The five different areas referenced in the survey were: (1) providing health care to animals in zoo care, (2) research on how diseases can threaten species with extinction, (3) studying diseases in animals in zoo care, (4) studying how diseases in wild animals have an impact on domestic animals, and (5) research comparing diseases between different species, including humans (Figure 1). Eighty-six percent of respondents were aware of the Zoo’s role in providing health care to animals in zoo care, 80% were interested, and 95% thought it was important. Fifty-five percent of respondents were aware of the Zoo’s role in research on how diseases can threaten species with extinction, 67% were aware of the Zoo’s role in studying diseases in animals in zoo care, but only 27% were aware of the Zoo’s role in studying how diseases in wild animals have an impact on domestic animals and humans, and only 23% were aware of the Zoo’s role in research comparing diseases between different species, including humans. For each of the five areas, except for the category of “providing health care to zoo animals in zoo care,” participants were consistently less aware of the Zoo’s role in each specific area than their interest and perceived importance of the Zoo’s work in that role. Individuals over the age of 55 were significantly more interested in the Zoo’s participation in these areas compared to the other age categories.

## Discussion

The findings from the survey revealed that Zoo members primarily cared about chronic, non-communicable diseases, and their associated costs. These results were initially surprising, because at the time of the survey, Zika outbreaks were receiving heavy media attention. However, when examined in the context of population surveyed and the political climate at the time, these results were more understandable. The United States has already undergone an epidemiologic transition and thus, more people are afflicted with chronic, non-communicable diseases that are associated with increased health-care costs. Leading up to the 2016 United States Presidential election, there was growing unrest in the general American population around the recent implementation of the Affordable Care Act, mostly aimed at the lack of affordability of health care. While the survey results indicated Zoo members focused on health-care costs, these results may not be representative of the larger American population. Possibly related to the physical geography of the United States and the varying population densities, there may be a higher perceived threat from zoonotic diseases in people residing in coastal areas compared to the Midwest. Although One Health initiatives by zoos and other conservation organizations have primarily focused on messaging about infectious, zoonotic diseases (11, 13) to more effectively promote One Health programs at zoos, it may be beneficial for zoos to address the health concerns more specific to their zoo-going population. The results of this study may serve as a template to help guide zoos in their development of One Health messaging.

Along with learning about human health benefits, Zoo members were overwhelmingly interested in learning about the Zoo’s activities in One Health and believed these to be an important role for the Zoo. People were more familiar with the term “Conservation Medicine” (16%) than “One Health” (6%), but their general lack of awareness of the terms themselves indicates that we should do more to effectively disseminate information about the ICM and the role of the Saint Louis Zoo in One Health.

The survey indicated that there is interest in One Health in the St. Louis community of Zoo members and to promote it messaging should include One Health in the context of chronic, non-communicable diseases, in addition to our other more ecological research-focused programming. This messaging should also be aimed at individuals over the age of 55, who were consistently interested in the Zoo’s activities, but less aware. People were generally aware of One Health activities, but then were unable to put a term to these activities. The messaging from the ICM about One Health, *via* newsletters, magazines, talks, and social media outlets did appear to have some impact on the member community. The ICM continues to pave the way for further more effective messaging to the Zoo’s membership and visitors regarding One Health.

This study provided a brief, but valuable insight into Zoo members’ understanding of One Health, which may help inform not only our future work at the ICM, but also at other zoos as they develop programs to promote and disseminate information regarding the One Health initiative. While the results obtained are valuable, it is important to note the limitations of the study itself. The surveyed population was Zoo members, who primarily reside in St. Louis and the surrounding areas. As the Saint Louis Zoo does not have an admission fee for visitors, its members may be distinct socioeconomically from the general population of Zoo visitors. Even with this distinction, the study population still represented a varied socioeconomic demographic, with 40% of study respondents being from zip codes below the median household income in the United States. The study is also subject to volunteer bias, as we sent the survey to over 3,000 individuals, but only 285 provided complete answers. In regards to how the respondents answered questions, the survey was distributed when there was heavy media attention on the Zika outbreak, which may have influenced responses to health concerns: however, our data do not support this line of thinking. Additionally, survey respondents primarily lived in urban/suburban areas, and thus the results are not generalizable to rural settings. Overall, this survey offered a valuable initial look into the minds of Zoo visitors/members, with results that may help assist other zoos in One Health program development and/or implementation.

On the survey, individuals identified poor diet/obesity/lack of nutrition as one of their top three concerns. This concern may be easily addressed by One Health messaging since zoos help tackle this challenge by efforts to protect pollinators, like bees and bats, thus increasing food security and enabling a higher quality diet. Zoos provide communities with access to greenspace, and it has been shown that increased time in nature may mitigate the negative health impacts of urbanization (17, 19, 20, 22, 26, 27). Zoos promote healthy lifestyles by providing access to exercise and requiring movement by visitors, thus potentially having an impact on obesity, and health-care costs (18). Zoos also play an important role in conservation, which has long-term health impacts for individuals, reduces health-care costs, and increases healthy behaviors (5, 27).

While individuals’ concerns over health-care costs and chronic diseases need to be addressed by One Health practitioners, infectious diseases should not be neglected in communication between zoos and the public. As climate change progresses and human habitats expand, there will be an increase in human–wildlife interactions, leading to an increase in zoonotic disease transmission (21, 28–30). Zoos have both the resources and capabilities to promote One Health, and they may provide messaging about it by sharing information on the positive health impacts zoos offer to their human visitors, while continuing to work to promote healthy habitats for humans and animals.

## Ethics Statement

This study involved an online survey of members and was exempt from Institutional Review Board (IRB) approval. Completion of the survey indicated consent.

## Author Contributions

Conception or design of the work and acquisition, analysis or interpretation of data for the work; drafting the work or revising it critically for important intellectual content; final approval of the version to be published: and agreement to be accountable for all aspects of the work: HP, AN, ET, and SD.

## Conflict of Interest Statement

The authors declare that the research was conducted in the absence of any commercial or financial relationships that could be construed as a potential conflict of interest.
